# Efficient population record linkage with temporal and spatial constraints.

**DOI:** 10.23889/ijpds.v7i3.1854

**Published:** 2022-08-25

**Authors:** Charini Nanayakkara, Peter Christen

**Affiliations:** 1 School of Computing, The Australian National University

## Objectives

Population databases containing birth, death, and marriage certificates or census records, are increasingly used for studies in a variety of research domains. Their large scale and complexity make linking such databases highly challenging. We present a scalable blocking and linking technique that exploits temporal and spatial constraints in personal data.

## Approach

Based on a state-of-the-art blocking method using locality sensitive hashing (LSH), we incorporate (a) attribute similarities, (b) temporal constraints (for example, a mother cannot give birth to two babies less than nine months apart, besides a multiple birth), and (c) spatial constraints (two births by the same mother are more likely to happen in the same location than far apart). In an iterative fashion, we identify highly confident matches first, and use these matches to further refine our constraints. We adopt a block size and frequency-based filtering approach to further enhance the efficiency of the record linkage comparison step.

## Results

We conducted experiments on a Scottish data set containing 17,613 birth certificates from 1861 to 1901, where the application of standard LSH blocking generated approximately 15 million candidate record pairs, with a recall of 0.999 and a precision of 0.003. With the application of our block size and frequency-based filtering approach we obtained a ten-fold and hundred-fold reduction of this candidate record pair set with a small reduction of recall to 0.984 and 0.962, respectively. The comparison of record pairs in the hundred-fold reduction using our iterative linking technique achieved up-to 0.961 precision and 0.811 recall. This means that our method can achieve a reduction in computational efforts, and improvement in precision of over 99% at the cost of a decline in recall below 19%.

## Conclusion

We presented a method to reduce the computational complexity of linking large and complex population databases while ensuring high linkage quality. Our method can be generalised to population databases where temporal and spatial constraints can be defined. We plan to apply our method on a Scottish database with 24 million records.

